# Interpretable Machine Learning Predictions of Bruch’s Membrane Opening-Minimum Rim Width Using Retinal Nerve Fiber Layer Values and Visual Field Global Indexes

**DOI:** 10.3390/bioengineering12030321

**Published:** 2025-03-20

**Authors:** Sat Byul Seo, Hyun-kyung Cho

**Affiliations:** 1 Department of Mathematics Education, Kyungnam University, 7 Kyungnamdaehak-ro, Changwon-si 51767, Geongsangnam-do, Republic of Korea; sbseo@kyungnam.ac.kr; 2 Department of Ophthalmology, Gyeongsang National University Changwon Hospital, School of Medicine, Gyeongsang National University, Changwon-si 51472, Geongsangnam-do, Republic of Korea; 3 lnstitute of Medical Sciences, School of Medicine, Gyeongsang National University, Jinju-si 52727, Geongsangnam-do, Republic of Korea

**Keywords:** BMO-MRW, Bruch’s membrane opening-minimum rim width, optical coherence tomography, visual field, VF global index, machine learning, gradient boosting regression, SHAP

## Abstract

The aim of this study was to predict Bruch’s membrane opening-minimum rim Width (BMO-MRW), a relatively new parameter using conventional optical coherence tomography (OCT) parameter, using retinal nerve fibre layer (RNFL) thickness and visual field (VF) global indexes (MD, PSD, and VFI). We developed an interpretable machine learning model that integrates structural and functional parameters to predict BMO-MRW. The model achieved the highest predictive accuracy in the inferotemporal sector (R^2^ = 0.68), followed by the global region (R^2^ = 0.67) and the superotemporal sector (R^2^ = 0.64). Through SHAP (SHapley Additive exPlanations) analysis, we demonstrated that RNFL parameters were significant contributing parameters to the prediction of various BMO-MRW parameters, with age and PSD also identified as critical factors. Our machine learning model could provide useful clinical information about the management of glaucoma when BMO-MRW is not available. Our machine learning model has the potential to be highly beneficial in clinical practice for glaucoma diagnosis and the monitoring of disease progression.

## 1. Introduction

Glaucoma arises from damage to retinal ganglion cells (RGCs) and their axons, resulting in deficits in the retinal nerve fibre layer (RNFL) and thinning of the neuroretinal rim (NRR), which may lead to visual field (VF) defects [1]. Quantitative evaluation of the structural damage in glaucoma is commonly performed through the measurement of peripapillary RNFL thickness using optical coherence tomography (OCT) [2]. Functional VF defects are identified and monitored using standard automated perimetry (SAP), which remains the gold standard in glaucoma management [3,4]. The relationship between structural and functional changes is critical for understanding and managing glaucoma [5,6,7,8]. Structural alterations are often detectable before VF functional loss in the early stages of the disease [8,9,10,11,12].

**Figure 1:**
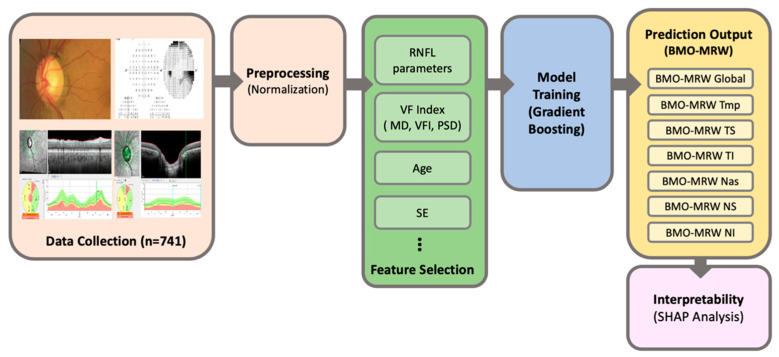
Workflow of machine learning model for predicting BMO-MRW parameters.

Spectral-domain optical coherence tomography (SD-OCT) has recently introduced Bruch’s membrane opening-minimum rim width (BMO-MRW) as a novel parameter, complementing the conventional measurement of peripapillary RNFL thickness. BMO-MRW represents the shortest distance between the inner edge of the Bruch’s membrane opening and the internal limiting membrane (Figure 1, the bottom right of the first box). It has also been proposed for the assessment of the optic nerve head [13,14,15,16,17]. This parameter provides a more precise evaluation of the NRR compared to traditional optic disc inspection [13,14,15,16,17,18]. Studies have demonstrated that BMO-MRW exhibits superior diagnostic performance in glaucoma compared to previously utilized NRR parameters [9,10,11,12,13,14,15,16,17,18,19,20,21]. Furthermore, BMO-MRW has been shown to exhibit a stronger structure–function correlation than other NRR measurements or peripapillary RNFL [21,22].

In our previous study, we analyzed the clinical characteristics of patients exhibiting discrepancies between BMO-MRW and peripapillary RNFL thickness in OCT [23]. We observed that in cases involving large optic discs and myopia—key features in many glaucoma suspect patients—RNFL parameters frequently indicated abnormalities, whereas BMO-MRW remained within normal limits. Importantly, VF testing in these cases also yielded normal results. This discrepancy can be attributed to the temporalization of RNFL peaks in myopic eyes and the nasalization of RNFL peaks in large optic discs, which can result in abnormal classifications of RNFL that are not reflected in BMO-MRW results [23]. This is partly because these two structural parameters are measured at different locations of the optic disc. BMO-MRW assesses NRR, while RNFL measures RNFL thickness around the optic disc.

These observations suggest that incorporating BMO-MRW measurements can provide valuable additional diagnostic information in distinguishing early glaucoma from glaucoma suspects, particularly when conventional RNFL findings yield false positives. The misinterpretation of RNFL abnormalities in conventional OCT could lead to overtreatment of glaucoma suspect patients, resulting in unnecessary costs and interventions. However, it is important to note that the BMO-MRW parameter is exclusively available in the Heidelberg Spectralis OCT Glaucoma Module Premium Edition (Heidelberg Engineering, Heidelberg, Germany).

Despite this limitation, clinicians could potentially improve early glaucoma diagnosis if additional information on BMO-MRW is available, and also by integrating existing RNFL parameters with visual field indices, such as mean deviation (MD), pattern standard deviation (PSD), and visual field index (VFI). This approach could be particularly beneficial in early-stage glaucoma, where diagnostic challenges are more pronounced due to the subtlety of structural and functional changes, in contrast to later stages where damage is extensive, and test results are uniformly abnormal [23].

It is difficult to find a previous study that predicted BMO-MRW using a deep learning model. However, one study predicted BMO-MRW values from optic disc photographs using a deep learning method of convolutional neural network (CNN) [24].

In our recently published study, we developed a deep learning model capable of predicting VF global indices based on OCT parameters, specifically BMO-MRW and RNFL thickness. The model demonstrated high predictive accuracy, achieving mean absolute errors (MAE) of 1.9–2.9 dB for MD, 1.6–2.0 dB for PSD, and 5.0–7.0% for the VFI [25]. Building on this prior experience, the present study aimed to develop a deep learning model to predict BMO-MRW using RNFL values and VF indices (MD, PSD, and VFI), which integrates both structural (RNFL) and functional (VF) parameters of glaucoma. We chose these parameters to predict BMO-MRW because RNFL is also a structural parameter and an existing parameter obtained by any devices of OCT, but is not quite the same. In this regard, we added VF global indices to compensate for the discrepancy between the two structural parameters and to add a functional parameter to reflect that BMO-MRW has strong structure–function relationship for glaucoma. Such a model could provide valuable supplementary clinical information for diagnosing glaucoma, particularly in cases where BMO-MRW measurements are unavailable, as not all OCT devices offer this parameter. By leveraging these predictions, clinicians could enhance diagnostic accuracy and optimize patient management, especially in the context of early glaucoma.

## 2. Materials and Methods

### 2.1. Ethics Statement

This retrospective, observational, and cross-sectional study was conducted in compliance with the principles outlined in the Declaration of Helsinki. Ethical approval was obtained from the Institutional Review Board (IRB) of Gyeongsang National University Changwon Hospital, affiliated with the Gyeongsang National University School of Medicine. Given the retrospective design of the study, the IRB waived the requirement for obtaining informed consent.

### 2.2. Subjects

Among 1528 patients with glaucoma or glaucoma suspects evaluated between February 2016 and December 2024 at the glaucoma clinic of Gyeongsang National University Changwon Hospital, a total of 741 eyes (741 subjects) were included in the study. The glaucoma diagnoses encompassed normal-tension glaucoma (NTG), primary angle-closure glaucoma (PACG), pseudoexfoliation glaucoma (PEX G), primary open-angle glaucoma (POAG), and glaucoma suspects (GS). The subjects consisted of 229 eyes from patients classified as GS, 308 eyes with NTG, 36 eyes with PACG, 63 eyes with PEX G, and 105 eyes with POAG. Only participants meeting the diagnostic criteria outlined below and demonstrating reliable results for both BMO-MRW and RNFL measurements were included.

The diagnosis of glaucoma was conducted by a single glaucoma specialist (H-K Cho) using standardized criteria. NTG diagnosis required intraocular pressure (IOP) ≤ 21 mmHg without treatment, glaucomatous optic disc damage with corresponding VF defects, an open-angle confirmed by gonioscopic examination, and the exclusion of other causes of optic disc damage [26]. PACG was diagnosed in eyes with a shallow anterior chamber, characterized by appositional contact between the peripheral iris and trabecular meshwork over >270° on gonioscopy, glaucomatous optic disc damage (evidenced by NRR thinning, vertical cup-to-disc ratio ≥ 0.7 or asymmetry ≥ 0.2 between eyes, or notching attributable to glaucoma), and corresponding VF defects [27]. For PEX glaucoma, diagnosis required the presence of pseudoexfoliative material on the anterior lens capsule and at the pupil margin following maximal pupil dilation, baseline IOP ≥ 22 mmHg, glaucomatous optic nerve head damage, VF defects consistent with optic disc injury, and the exclusion of other causes of secondary glaucoma [28]. POAG was defined as baseline IOP > 21 mmHg (prior to treatment), glaucomatous optic nerve head damage with corresponding VF loss, an open-angle confirmed by gonioscopy, and no other underlying cause for optic nerve head damage besides glaucoma [1].

The exclusion criteria for the study were as follows: low-quality imaging scans due to factors such as eyelid blinking or poor fixation; a history of optic neuropathies other than glaucoma or acute angle-closure crises that could influence RNFL or BMO-MRW thickness (e.g., optic neuritis, acute ischemic optic neuropathy); a history of any intraocular surgery, except for uncomplicated phacoemulsification; and retinal diseases associated with retinal swelling or edema that could cause subsequent thickening of the RNFL or BMO-MRW. Additionally, cases of preperimetric glaucoma were excluded from the analysis. However, axial length, refractive error, and optic disc size were not considered exclusion criteria in the present study.

### 2.3. Optical Coherence Tomography

SD-OCT imaging was performed using the Glaucoma Module Premium Edition. A total of 24 radial B-scans were obtained to evaluate the BMO-MRW. For peripapillary RNFL thickness measurements, a scan circle diameter of 3.5 mm was selected from the available options (3.5, 4.1, and 4.7 mm). Only images that were properly centred, accurately segmented, and had quality scores ≥ 20 were included in the study. The OCT images were aligned using the fovea-to-Bruch’s membrane opening (FoBMO) axis, an individual-specific axis defined by the line connecting the centre of the BMO and the foveal centre. Utilizing the FoBMO axis allowed for more accurate analysis of the Garway-Heath sector by accounting for individual cyclotorsion, and provided a more precise comparison with the normative database than the conventional approach based solely on clock-hour locations.

### 2.4. Perimetry

Perimetry was conducted using a Humphrey Field Analyzer (HFA model 840; Humphrey Instruments Inc., San Leandro, CA, USA) with the central 30-2 programme and the Swedish Interactive Threshold Algorithm (SITA) standard strategy. A VF test was considered reliable if it met the following criteria: a false-positive rate of <15%, a false-negative rate of <15%, and a fixation loss of <20%.

### 2.5. Data Preprocessing

The dataset included OCT-derived parameters for 741 eyes, comprising age, spherical equivalent (SE), BMO-MRW parameters (Global, Temporal (T), Superotemporal (TS), Inferotemporal (TI), Nasal, Superonasal (NS), and Inferonasal (NI)), RNFL parameters (Global, Temporal, TS, TI, Nasal, NS, and NI), and visual field (VF) indices (Mean Deviation (MD), Pattern Standard Deviation (PSD), and Visual Field Index (VFI)). The dataset was structured into target columns (BMO-MRW parameters) and feature columns (remaining clinical and RNFL parameters). The data were randomly split into a training set (80%, 592 eyes) and a test set (20%, 149 eyes), followed by normalization using StandardScaler from Scikit-learn to ensure a standardized feature scale across variables. All preprocessing steps, including data handling, normalization, and splitting, were implemented using Python version 3.11.11 (https://www.python.org/, accessed on 20 March 2025) and Scikit-learn version 1.6.0 (https://scikit-learn.org/, accessed on 20 March 2025).

### 2.6. Machine Learning Algorithm

Gradient boosting is an ensemble supervised machine learning algorithm that can be used for both classification and regression problems. Gradient Boosting Decision Trees (GBDT) operate by sequentially constructing weak learners—typically decision trees—where each new tree corrects the residual errors of the previous trees. Given input features X and output targets Y, the gradient boosting framework minimizes a differentiable loss function $L\left(Y,\;F\left(X\right)\right)$ iteratively:

$$F_{m}\left(X\right)=F_{m-1}\left(X\right)+\gamma_{m}h_{m}\left(X\right),$$

where $F_{m}\left(X\right)$ is the boosted model at iteration m, $h_{m}\left(X\right)$ is a weak learner (decision tree) trained on residual, and $\gamma_{m}$ is the learning rate optimizing the loss function. The foundational principle of GBR is that it optimizes an objective function using a gradient descent approach in function space, refining predictions iteratively to minimize residual errors [29]. GBDT has been found to outperform traditional regression methods, such as logistic regression, particularly when handling large-scale datasets [30]. Gradient Boosting Decision Trees (GBDT) are particularly effective for small datasets and frequently demonstrate superior performance compared to other methods such as deep learning [31]. We applied the Multi-Output Gradient Boosting Regressor (MGBR), an extension of Gradient Boosting Regression Trees (GBRT) which leverages gradient boosting to predict multiple BMO-MRW parameters simultaneously. For multi-output regression, the objective function extends to the following:

$$L\left(Y,\;F\left(X\right)\right)=\overset{K}{\underset{k=1}{\sum }}\overset{n}{\underset{i=1}{\sum }}L(y_{i,k}\;,\;F_{k}\left(X_{i}\right)),$$

where K is the number of target variables, ensuring simultaneous optimization of multiple BMO-MRW parameters. This approach enabled us to effectively capture complex, nonlinear relationships while concurrently estimating multiple BMO-MRW parameters [32]. MultiOutputRegressor with GradientBoostingRegressor is available in scikit-learn.

### 2.7. Workflow of Machine Learning Model for Predicting BMO-MR

We aimed to predict the parameters of BMO-MRW: BMO-MRW Global, BMO-MRW Temporal, BMO-MRW TS, BMO-MRW TI, BMO-MRW Nasal, BMO-MRW NS, and BMO-MRW NI from the parameters of RNFL, age, SE, and three VF global indices (MD, PSD, and VFI) based on machine learning. The main workflow of the machine learning model for predicting BMO-MRW is as follows: First, numerical parameters of BMO-MRW and RNFL were extracted from the OCT scan images of patients, along with clinical parameters such as age, SE, and VF global indices. Preprocessing steps involved handling normalizing input parameters. A total of 741 eyes from 741 patients were included in the analysis. Gradient Boosting was employed as the machine learning model, with 5-fold cross-validation applied to optimize hyperparameters such as the learning rate (0.1), number of trees, (*n* = 100) and maximum depth of each tree (3). The dataset was divided into training and test sets, and the model’s performance was assessed on the test set by predicting each BMO-MRW parameter. To enhance interpretability, SHAP analysis was used to understand the contribution of each input feature to the model’s predictions. The workflow is illustrated in Figure 1, which includes the preprocessing, feature selection, model training, and interpretability steps.

### 2.8. Statistical Analysis

To evaluate the performance of the gradient boosting regression model, Mean Absolute Error (MAE) and Mean Squared Error (MSE) were utilized. MAE was assessed to measure the interpretability and accuracy of the regression model, as it provides an intuitive understanding of prediction errors by averaging the absolute differences between actual and predicted values. MSE has the advantage of being more sensitive to outliers due to the squared penalty on residuals. The formulas for MAE and MSE are as follows:

$$\mathrm{MAE}=\frac{1}{n}\overset{n}{\underset{i=1}{\sum }}\left|y_{i}-\hat{y_{i}}\right|,$$

$$\mathrm{MSE}=\frac{1}{n}\overset{n}{\underset{i=1}{\sum }}{\left(y_{i}-\hat{y_{i}}\right)}^{2},$$

where $y_{i}$ represents the actual value, $\hat{y_{i}}$ is the predicted value, and n is the total number of samples.

We also computed Pearson’s correlation coefficient (ρ) to assess the relationships among all key variables, including explanatory (RNFL values, Age, SE, and VF global indices) and target variables (BMO-MRW parameters) and $R^{2}$ to assess the effectiveness of model training and the reliability of its predictions [33]. The formulas for ρ and $R^{2}$ are as follows.

$$\rho =\frac{\sum_{i=1}^{n}\left(x_{i}-\bar{x\;}\right)\left(y_{i}-\bar{y\;}\right)}{\sqrt{\sum_{i=1}^{n}{\left(x_{i}-\bar{x\;}\right)}^{2}{\left(y_{i}-\bar{y\;}\right)}^{2}}},\;\;\;\;\;\;\bar{x}:\;\mathrm{mean}\;\mathrm{of}\;x,\;\;\;\bar{y}:\;\mathrm{mean}\;\mathrm{of}\;y,$$

$$R^{2}=1-\frac{\sum {\left(y_{i}-\hat{y_{i}}\right)}^{2}}{\sum {\left(y_{i}-\bar{y}\right)}^{2}},\;\;\;\;\;\;\;\;\;\;\;\;\;\;\;\;\;\;\;\;\;\hat{y_{i}}:\;\mathrm{estimation}\;\mathrm{of}\;y_{i},\;\;\;\bar{y}:\;\mathrm{mean}\;\mathrm{of}\;y,$$

$x_{i}$ and $y_{i}$ represent any two selected variables among the explanatory variables (RNFL values, Age, SE, and VF global indices) and target variables (BMO-MRW parameters). All statistical analyses were performed using programming language Python version 3.11.11 (https://www.python.org/) and Scikit-learn package version 1.6.0 (https://scikit-learn.org/).

### 2.9. SHAP (SHapley Additive exPlanations)

SHAP (SHapley Additive exPlanations) is a game-theoretic framework for interpreting machine learning predictions by quantifying the importance of each feature for a specific prediction. Although complex models, such as ensemble methods and deep learning, often achieve superior accuracy on large-scale modern datasets, their inherent lack of interpretability presents a considerable trade-off. SHAP bridges the concept of optimal credit allocation with local interpretability by leveraging the classic Shapley values from game theory and their related extensions [34]. For a given feature i, the SHAP value $\emptyset_{i}$ is defined as:

$$\emptyset_{i}=\sum_{S\;⊑\;N\backslash \left\{i\right\}}\frac{\left|S\right|!\left(\left|N\right|-\left|S\right|-1\right)!}{\left|N\right|!}\left[f_{S\cup \left\{i\right\}}\left(x\right)-f_{S}\left(x\right)\right],$$

where $\emptyset_{i}$ is the contribution of feature i to the model’s prediction. N represents the set of all features, f is the predictive model. $N\backslash \left\{i\right\}$ denotes the set of all features except for feature i. $\left|N\right|,\;\left|S\right|$ are the sizes of the subset N, S respectively. $\frac{\left|S\right|!\left(\left|N\right|-\left|S\right|-1\right)!}{\left|N\right|!}$ represents the weight assigned to each subset based on its size. The tree SHAP we applied is a specialized algorithm designed for interpreting tree-based models, such as Random Forests and Gradient Boosting Trees. For a tree-based model, it is sufficient to calculate SHAP values on a single tree, as the SHAP values for an ensemble of trees are simply the sum of the SHAP values from each individual tree according to the additivity property of SHAP values. It is defined as a conditional expectation $f_{S}\left(x\right)=E[f\left(x\right)|\;x_{s}]$ for the tree-based model [34,35,36]. SHAP not only unifies existing feature attribution methods but also provides a unique solution for additive feature importance measures, enabling interpretable and consistent explanations across a wide range of machine learning models [35].

## 3. Results

### 3.1. Baseline Characteristics of the Dataset

A total of 741 eyes from 741 patients with glaucoma were included in the final analysis. The glaucoma diagnoses encompassed NTG, PACG, PEX G, POAG, and glaucoma suspects. The mean age of the dataset was 53.62 ± 13.53 years (mean ± standard deviation), with 45.21% (335/741) being female and 8.1% (60/741) reporting a family history of glaucoma. Baseline spherical equivalent (SE) was −1.67 ± 3.30 diopters, while baseline IOP averaged 15.51 ± 4.14 mmHg. Central corneal thickness (CCT) was recorded as 542.43 ± 42.70 µm. The baseline VF global indices were as follows: MD was −5.74 ± 35.11 dB, PSD was 5.29 ± 4.16 dB, and VFI was 88.37 ± 12.29 dB. These findings, including RNFL and BMO-MRW parameters, are summarized in Table 1.

### 3.2. Prediction Performance a Gradient Boosting Model

The predictive performance of the gradient boosting regression model was evaluated across seven BMO-MRW targets using three metrics: MAE, MSE, and the coefficient of determination (R^2^), based on a test set comprising 149 eyes. The *R^2^* values for BMO-MRW Global, BMO-MRW TS, BMO-MRW TI, and BMO-MRW NI were 0.67, 0.64, 0.68, and 0.70, respectively, indicating strong predictive accuracy for these targets. Conversely, the R^2^ values for BMO-MRW Tmp, BMO-MRW Nas, and BMO-MRW NS were comparatively lower at 0.46, 0.40, and 0.54, respectively. Among the targets, BMO-MRW Global demonstrated the highest overall performance, achieving an *R^2^* value of 0.67, along with the lowest MAE and MSE values, highlighting its superior predictive reliability. These findings are summarized in Table 2.

**Figure 2:**
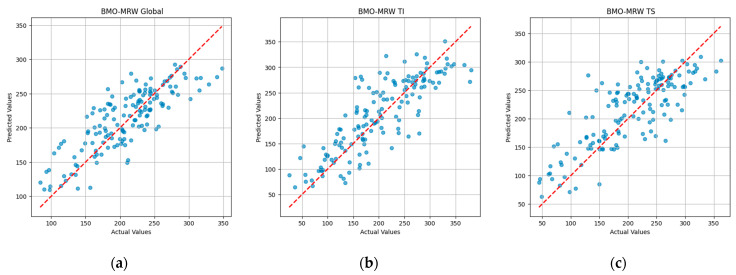
Scatter plots illustrating the comparison between predicted and actual values for three BMO-MRW parameters: (**a**) BMO-MRW Global, (**b**) BMO-MRW TI, and (**c**) BMO-MRW TS. The red dashed diagonal line represents y=x, indicating exact match between predicted and actual values.

Furthermore, we evaluated the gradient boosting regression model’s predictive performance for the seven BMO-MRW parameters by visually assessing the alignment between predicted and actual values. Figure 2 presents scatter plots illustrating the comparison between predicted and actual values for three BMO-MRW parameters: BMO-MRW Global, BMO-MRW TI, and BMO-MRW TS. These parameters, clinically significant sectors, indicate strong predictive performance based on their R^2^ values exceeding 0.6 and their relatively low MAE and MSE values. The scatter plots provide a visual representation of the model’s accuracy, with data points closer to the y=x diagonal line reflecting a higher match between predictions and actual values. This visualization provides an intuitive representation of the model’s accuracy for each parameter. The results for all BMO-MRW parameters are provided in the Supplementary Materials.

### 3.3. Correlation Analysis and Interpretable Machine Learning

**Figure 3:**
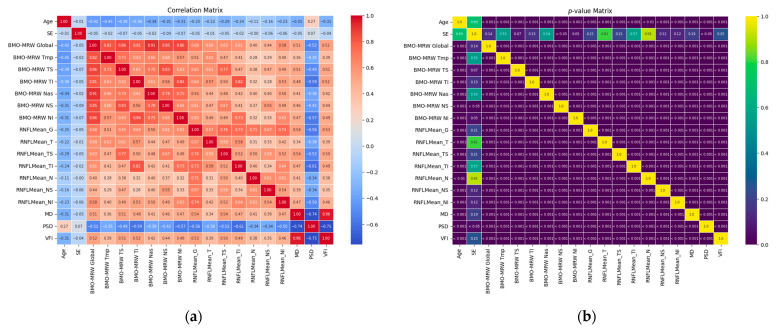
Heatmaps of (**a**) Correlation Coefficients and (**b**) *p*-values Between Feature and target Parameters (Age, SE, RNFL parameters, and BMO-MRW parameters).

To examine the relationships between the BMO-MRW parameters and the feature parameters, we analyzed their pairwise correlations and statistical significance. Figure 3a,b presents the heatmaps of correlation coefficients and their corresponding *p*-values, respectively, between the feature parameters (Age, SE, and RNFL parameters) and the target parameters (BMO-MRW parameters). As shown in Figure 3a, the target BMO-MRW parameters generally exhibit notable correlations with Age and RNFL parameters, while SE shows weak correlation. RNFL G demonstrates a high correlation with most BMO-MRW parameters, with correlation coefficients generally approaching or exceeding 0.6, except for BMO-MRW Tmp (0.53) and BMO-MRW Nas (0.56), which are slightly below this threshold. RNFL TI and BMO-MRW TI have the highest correlation coefficient of 0.82, followed by RNFL TS and BMO-MRW TS with a coefficient of 0.77. Additionally, Age and PSD exhibit negative correlations with all target parameters, indicating an inverse relationship. Figure 3b shows the corresponding *p*-values, which confirm the statistical significance of most correlations, reflecting strong and reliable associations with the BMO-MRW targets.

**Figure 4:**
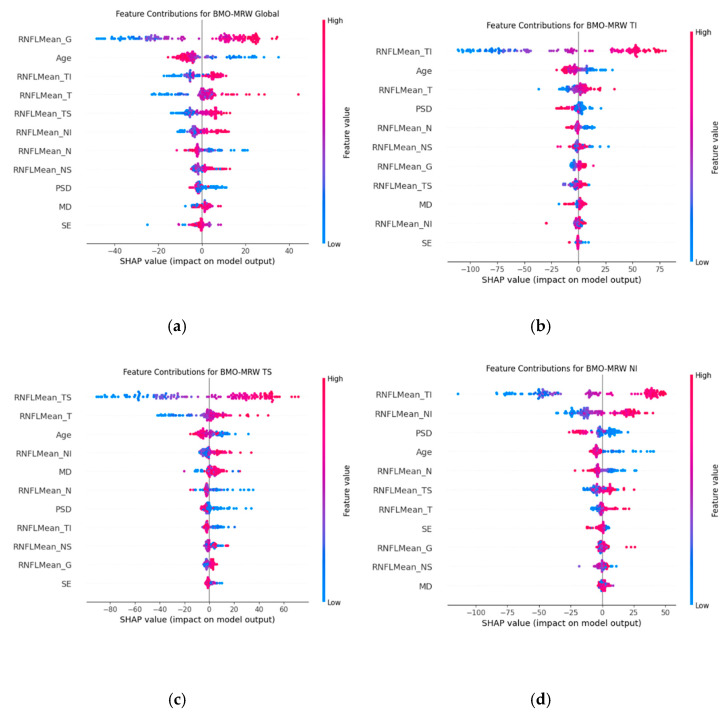
SHAP (SHapley Additive exPlanations) Summary Plots for Feature Contributions to the Prediction of (**a**) BMO-MRW Global, (**b**) BMO-MRW TI, (**c**) BMO-MRW TS, (**d**) BMO-MRW NI. These plots demonstrate the impact of individual features on the model output, where the *x*-axis represents the SHAP values indicating the magnitude and direction of feature influence, and the colour gradient denotes feature values from low (blue) to high (red).

To evaluate the performance of the machine learning model in predicting BMO-MRW parameters, we further analyzed which features significantly contributed to the prediction of each parameter. To ensure reliable feature attribution, we addressed the concern regarding multicollinearity in SHAP analysis by removing features with correlation coefficients above 0.8. Specifically, VFI and MD had a high correlation of 0.96, so VFI was removed. Using SHAP, we generated summary plots to identify the most influential features for each prediction. These plots demonstrate the impact of individual features on the model output, where the x-axis represents the SHAP values indicating the magnitude and direction of feature influence, and the colour gradient denotes feature values from low (blue) to high (red). Figure 4 shows the SHAP summary plots for feature contributions to the prediction of BMO-MRW Global, BMO-MRW TI, BMO-MRW TS, and BMO-MRW NI. In Figure 4a, RNFL G, Age, and RNFL TI are identified as the most influential features contributing to the prediction of BMO-MRW Global, highlighting their importance in the model’s output. Similarly, Figure 4b shows that RNFL TI, Age, and RNFL T significantly contribute to the prediction of BMO-MRW TI. Figure 4c demonstrates that RNFL TS, RNFL T, and Age are the critical features influencing the prediction of BMO-MRW TS. Lastly, Figure 4d shows that RNFL TI, RNFL NI, and PSD are the critical features influencing the prediction of BMO-MRW NI. The results for all BMO-MRW parameters are provided in the Supplementary Materials.

## 4. Discussion

To our knowledge, the present study was the first to predict BMO-MRW, a relatively new parameter of OCT from previous parameter of OCT, RNFL, and all three VF indexes including MD, PSD, and VFI. We developed a machine learning model based on both structural (RNFL) and functional (VF) parameters for the prediction of BMO-MRW and found that the performance was remarkable. Our model showed the best predictive performance for the inferotemporal sector (R^2^ = 0.68), followed by global region (R^2^ = 0.67) and superotemporal sector (R^2^ = 0.64), except for the inferonasal sector (R^2^ = 0.70). The predicted values and the actual values of BMO-MRW showed great correlation in the current study.

The utilization of Bruch’s membrane opening-minimum rim width (BMO-MRW) measurements obtained via spectral-domain optical coherence tomography (SD-OCT) has increased among clinicians, offering distinct advantages over traditional morphometric optic nerve head analyses performed with confocal scanning laser tomography [19,20,21]. Compared to conventional ophthalmic examination methods, BMO-MRW enables a more precise geometric evaluation of the NRR [13,14,15,18]. Notably, BMO-MRW provides an accurate representation of the neural tissue volume within the optic nerve [37].

A prior study of ours demonstrated the high diagnostic efficacy of BMO-MRW in differentiating early NTG from GS using a deep learning model based on OCT parameters, including BMO-MRW, peripapillary RNFL, and RNFL colour-coded classification (area under the curve [AUC]: 0.966) [38]. Interestingly, BMO-MRW alone exhibited superior diagnostic performance (AUC: 0.959) compared to RNFL thickness alone (AUC: 0.914) or RNFL with its colour-coded classification (AUC: 0.934). Moreover, the diagnostic performance of BMO-MRW as a single parameter was comparable to that of a combination of all three OCT parameters. These findings indicate that BMO-based optic disc assessment may offer a more comprehensive evaluation of the optic disc in glaucoma diagnosis compared to traditional methods. BMO-MRW appears clinically valuable in glaucoma diagnosis, potentially surpassing conventional RNFL metrics. The integration of BMO-MRW and RNFL assessments could further enhance diagnostic accuracy for glaucoma.

BMO-MRW is not only useful in the diagnosis of glaucoma but also in the monitoring the progression of the disease. Since the baseline value of BMO-MRW is greater than that of RNFL, it may show greater change in the progression of glaucoma than RNFL. In percentage, for example, 10% would be 21 μm for BMO-MRW and 8 μm for RNFL in the global region, respectively, with regard to Table 1 in this present study. In the previous study of ours, BMO-MRW exhibited a significantly greater rate of change compared to RNFL in eyes with disc hemorrhage (DH), particularly in terms of percentage reduction in the inferotemporal and superotemporal sectors [39]. These results of our study suggest that BMO-MRW may be more advantageous than RNFL for detecting glaucomatous progression at an earlier stage in eyes with DH, which are at a higher risk of progression [38].

However, not all OCT devices provide the parameter of BMO-MRW, and it is only available in Heidelberg Spectralis OCT Glaucoma Module Premium Edition (Heidelberg Engineering, GmbH, Germany). Therefore, the availability of this new parameter, BMO-MRW, although very useful, is limited for clinicians for the diagnosis and monitoring of glaucoma in the general setting. The integration of advanced artificial intelligence technology has the potential to significantly enhance clinical practice. Recent studies suggest that machine-learning classifiers can support general ophthalmologists in primary eye care settings, particularly in environments where glaucoma specialists are unavailable, by improving the efficiency and accuracy of glaucoma diagnosis [40]. Machine learning models can deliver rapid diagnostic results in clinical settings by analyzing ophthalmic examination data in real time, eliminating the need for multi-day analysis. While the ultimate decision to initiate glaucoma treatment remains the responsibility of the physician, machine learning models can provide a preliminary clinical result to aid clinical decision-making [41]. Furthermore, machine learning models offer a cost-effective and clinically accessible alternative to imaging-based convolutional neural network (CNN) diagnostic programmes, which often require expensive equipment, such as GPU-equipped workstations, and extended processing times to generate results.

Previous studies predicting BMO-MRW using artificial intelligence are rare to find, partly due to the relative novelty of this parameter. To our knowledge, the only related study is by Thompson et al., which used an imaging-based convolutional neural network (CNN) to predict BMO-MRW values from optic disc photographs [24]. In contrast, our study employs a machine learning approach based on Gradient Boosting Regression (GBR) using RNFL parameters and VF indices, integrating the structural and functional aspects of glaucoma. Gradient Boosting Decision Trees (GBDT) are particularly effective for small datasets and frequently outperform deep learning models in such cases [30]. Thompson et al. developed a deep learning algorithm to predict and quantify BMO-MRW from optic disc photographs. They used residual deep neural network (ResNet34) architecture for the prediction of BMO-MRW values, which is an imaging-based convolutional neural network (CNN). They included 743 eyes for the analysis, which is comparable to our study. We included 741 eyes for the final analysis. They also showed a great prediction performance with the best performance to be global region of R^2^ being 77%. Our study showed 0.67 as R^2^, which is 67%, a bit lower than their study. However, our study showed better prediction performance in the inferotemporal (R^2^ = 0.68) and superotemporal (R^2^ = 0.64) sectors than their study, which were 66% in the inferotemporal sector and 62% in the superotemporal sector, respectively. Unexpectedly, inferonasal sector showed the best performance in our study with R^2^ = 0.70. The prediction performance of the nasal sector in Thompson’s study revealed R^2^ of 68%, which was the best among six sectors, which is comparable to our study. It seems that the prediction of the nasal sector of BMO-MRW is higher than we thought. In our study, we assume that the inferonasal sector also could have served as one of the inferior sectors, which are more vulnerable to glaucomatous damage [42,43,44,45]. On the other hand, the nasal sector is not usually affected by glaucomatous injury at an early stage, and thus, the nasal sector may not show much change. Therefore, it could be easier to predict the nasal sector due to its stability of the values among enrolled subjects. This could be one of the explanations that the nasal sector and the inferonasal sector showed higher predictability among other sectors in Thompson’s study and also in the current study. These comparisons highlight that our proposed GBR-based approach is competitive and offers advantages in specific clinically relevant regions. Furthermore, we highlight the interpretability of our model through SHAP analysis, which deep learning models often lack.

Among the six Garway-Heath sectors of the OCT, the inferotemporal and superotemporal sectors are important in glaucoma. The site of initial glaucomatous injury is closely associated with the structural properties of the lamina cribrosa, which is widely recognized as the primary location of glaucomatous damage [43,44,45]. Early glaucomatous injury typically manifests in the inferior and superior portions of the axons [46], as the larger solitary-pore areas within the lamina cribrosa increase susceptibility to glaucomatous damage, particularly in the inferior region, followed by the superior optic disc area [47,48]. Due to this structural vulnerability, glaucomatous changes predominantly occur in the inferotemporal sector, followed by the superotemporal sector, during the early stages of the disease. Consequently, the inferotemporal sector plays a critical role in the diagnosis of early glaucoma and demonstrates superior diagnostic performance compared to other sectors.

Bowd et al. investigated the rate of change in BMO-MRW in different races of European and African descent. They have shown that changes in BMO-MRW predominantly occur in the temporal and inferior regions across all diagnostic groups of healthy, glaucoma suspect, and glaucoma [49]. The present study also showed that inferotemporal and superotemporal sectors of RNFL was important in predicting corresponding BMO-MRW values as analyzed by SHAP (Figure 3).

VF indexes also influenced the performance of predicting BMO-MRW values by our machine learning model. According to SHAP analysis, for the inferotemporal sector, VFI contributed as one of the high ranked factors, followed by PSD. And for the superotemporal sector, PSD was ranked as one of the contributing factors, followed by MD. Since BMO-MRW has demonstrated a stronger structure–function relationship compared to other NRR measurements or peripapillary RNFL [11,21], functional parameters of VF indexes may have contributed to the prediction of BMO-MRW value. Among VF indexes, PSD reflects earlier localized changes in glaucomatous VF defect, while MD is useful to estimate the overall stage of glaucoma [50]. In that regard, PSD has been ranked higher than MD in the results of the present study. Because using localized VF defect parameter could be more impactful to predict the actual value of sectoral BMO-MRW than generalized VF defect parameter.

Age was also an important contributing factor in the prediction of BMO-MRW values in our study as shown by SHAP analysis in all global regions, inferotemporal, superotemporal, and in the nasal sector (Figure 4). It is widely regarded that RNFL decreases according to age. It has also been reported that BMO-MRW also decreases with normal ageing [51]. The age-related loss of BMO-MRW should be carefully considered in estimating rates of change [51]. Since baseline RNFL and BMO-MRW values are affected by the age of the subject, age may have played a role in the prediction of BMO-MRW in the current study.

NTG accounts for the majority (76.3%) of POAG cases in Asian populations, as reported by previous population-based studies [52]. This highlights the clinical importance of understanding NTG, particularly in Asian countries and regions with a significant proportion of Asian populations. However, NTG has been underrepresented in prior deep-learning or machine learning studies, with few studies including NTG data. Given the scarcity of such studies, the current research contributes valuable information to the literature and may serve as a foundation for future deep-learning or machine learning studies in the field of glaucoma.

The current study has several limitations. First, its retrospective design introduces potential biases. Participants were limited to those who had undergone both RNFL and BMO-MRW tests with acceptable image quality, as well as reliable VF tests. The influence of this subject selection on the results remains uncertain. Second, the study was conducted within a referral university hospital in the province, employing a hospital-based design rather than a population-based approach, which may limit the generalizability of the findings. The individuals included in this study may not represent a fully generalizable sample of the broader population. Furthermore, the study was limited to Korean patients, meaning that the findings, including those related to NTG, may not be directly applicable to other ethnic groups.

Another limitation is the relatively small sample size. Although 741 subjects with either glaucoma or glaucoma suspect were included, this may not be insufficient to adequately train or test the predictive performance for a single test result derived from a single device’s data. Unlike larger studies that have utilized both eyes from multiple visits, we restricted our analysis to one randomly selected eye per participant from a single visit. While this approach may have reduced data volume (potentially increasing it sixfold had both eyes and multiple visits been included), it likely provided more independent and reliable results compared to previous studies that included large numbers of subjects.

Additionally, the analysis of OCT images in this study utilized numerical data extracted from the images, rather than direct image analysis. Despite this, the approach remains clinically meaningful as it enables the use of machine learning models with free open-source tools to provide rapid diagnostic support for glaucoma management. This method is also more cost-effective compared to convolutional neural networks (ConvNets), which require significant resources to achieve high accuracy in image analysis.

## 5. Conclusions

In conclusion, our machine learning model demonstrated high performance in predicting BMO-MRW values using OCT-derived parameter of RNFL thickness and VF indexes such as MD, PSD, and VFI. Our model predicted a relatively new parameter, BMO-MRW by integrating existing both structural parameter of RNFL and functional parameter of VF indexes. The prediction accuracy for BMO-MRW values was highest in the inferotemporal sector, followed by the global region and the superotemporal sector, with the exception of the inferonasal sector. Our machine learning model has the potential to be highly beneficial in clinical practice for managing glaucoma, including its diagnosis and monitoring of disease progression. The model’s ability to deliver prompt outputs makes it particularly valuable in primary eye care settings where glaucoma specialists may not be readily available. However, a more definitive assessment of its clinical utility would require a larger, multi-centre study with a substantial and diverse patient cohort.

## Figures and Tables

**Table 1:** Baseline characteristics.

Characteristics	Mean ± Std
Number of subjects	741 eyes (741 patients)
Mean Age (year)	53.62 ± 13.53
Female gender (%)	45.21 (335/741)
Family history of glaucoma (%)	8.1 (60/741)
Diagnosis	
NTG	308
POAG	105
PEX G	63
PACG	36
Glaucoma suspect	229
Spherical equivalent (D)	−1.67 ± 3.30
CCT (um)	542.43 ± 42.70
Baseline IOP (mmHg)	15.51 ± 4.14
MD (dB)	−5.74 ± 35.11
PSD (dB)	5.29 ± 4.16
VFI (%)	88.37 ± 12.29
RNFL Global	85.08 ± 21.40
RNFL Tmp	69.81 ± 17.43
RNFL TS	113.37 ± 37.63
RNFL TI	111.51 ± 46.90
RNFL Nas	68.15 ± 18.28
RNFL NS	100.29 ± 30.94
RNFL NI	93.09 ± 28.94
BMO-MRW Global	215.10 ± 58.44
BMO-MRW Tmp	167.16 ± 48.05
BMO-MRW TS	212.46 ± 74.42
BMO-MRW TI	214.36 ± 86.38
BMO-MRW Nas	233.17 ± 67.92
BMO-MRW NS	242.01 ± 73.53
BMO-MRW NI	249.83 ± 82.15

NTG = normal tension glaucoma; POAG = primary open-angle glaucoma; PEX G = pseudoexfoliation glaucoma; PACG = primary angle closure glaucoma; D = diopters; CCT = central corneal thickness; IOP = intraocular pressure; MD = mean deviation; PSD = pattern standard deviation; VFI = visual field index; RNFL = retinal nerve fibre layer; Tmp = temporal; TS = superotemporal; TI = inferotemporal; Nas = nasal; NS = superonasal; NI = inferonasal; BMO-MRW = Bruch’s membrane opening-minimum rim width.

**Table 2:** Prediction performance of a gradient boosting model.

Targets	MAE	MSE	*R* ^2^
BMO-MRW Global	25.17	1007.74	0.67
BMO-MRW Tmp	27.95	1278.75	0.46
BMO-MRW TS	34.42	1876.91	0.64
BMO-MRW TI	34.91	1999.98	0.68
BMO-MRW Nas	38.77	2403.80	0.40
BMO-MRW NS	35.94	2100.00	0.54
BMO-MRW NI	34.08	1889.31	0.70

BMO-MRW = Bruch’s membrane opening-minimum rim width; Tmp = temporal; TS = superotemporal; TI = inferotemporal; Nas = nasal; NS = superonasal; NI = inferonasal.

## Data Availability

The dataset utilized in this study might be obtained from Hyun-Kyung Cho (MD, PhD) upon reasonable request.

## Supplementary Materials

The following supporting information can be downloaded at: https://www.mdpi.com/article/10.3390/bioengineering12030321/s1, Figure S1: Scatter plots illustrating the comparison between predicted and actual values for the BMO-MRW parameters; Figure S2: SHAP (SHapley Additive exPlanations) Summary Plots for Feature Contributions to the Prediction of BMO-MRW parameters.

## Abbreviations

The following abbreviations are used in this manuscript:

RGCs	retinal ganglion cells
RNFL	retinal nerve fibre layer
NRR	neuroretinal rim
VF	visual field
OCT	optical coherence tomography
SAP	standard automated perimetry
SD-OCT	Spectral-domain optical coherence tomography
BMO-MRW	Bruch’s membrane opening-minimum rim width
MD	mean deviation
PSD	pattern standard deviation
VFI	visual field index
CNN	convolutional neural network
MAE	mean absolute errors
NTG	normal tension glaucoma
PACG	primary angle closure glaucoma
PEX G	pseudoexfoliation glaucoma
POAG	primary open-angle glaucoma
GS	glaucoma suspect
IOP	intraocular pressure
FoBMO	fovea-to-Bruch’s membrane opening
SITA	Swedish Interactive Threshold Algorithm
T	Temporal
TS	superotemporal
TI	inferotemporal
NS	superonasal
NI	inferonasal
GBR	Gradient Boosting Regression
GBDT	Gradient Boosting Decision Trees
MAE	Mean Absolute Error
MSE	Mean Squared Error
SHAP	SHapley Additive exPlanations
SE	spherical equivalent
CCT	Central corneal thickness
