# Supplementary material for: Interpretable Machine Learning Predictions of Bruch’s Membrane Opening-Minimum Rim Width Using Retinal Nerve Fiber Layer Values and Visual Field Global Indexes
Source: Bioengineering (Basel). 2025 Mar 20;12(3):321. doi: 10.3390/bioengineering12030321 (PMC11939392; doi:10.3390/bioengineering12030321)
Supplement: Supplementary file 1 [file bioengineering-12-00321-s001.zip › bioengineering-3480337-supplementary.pdf]

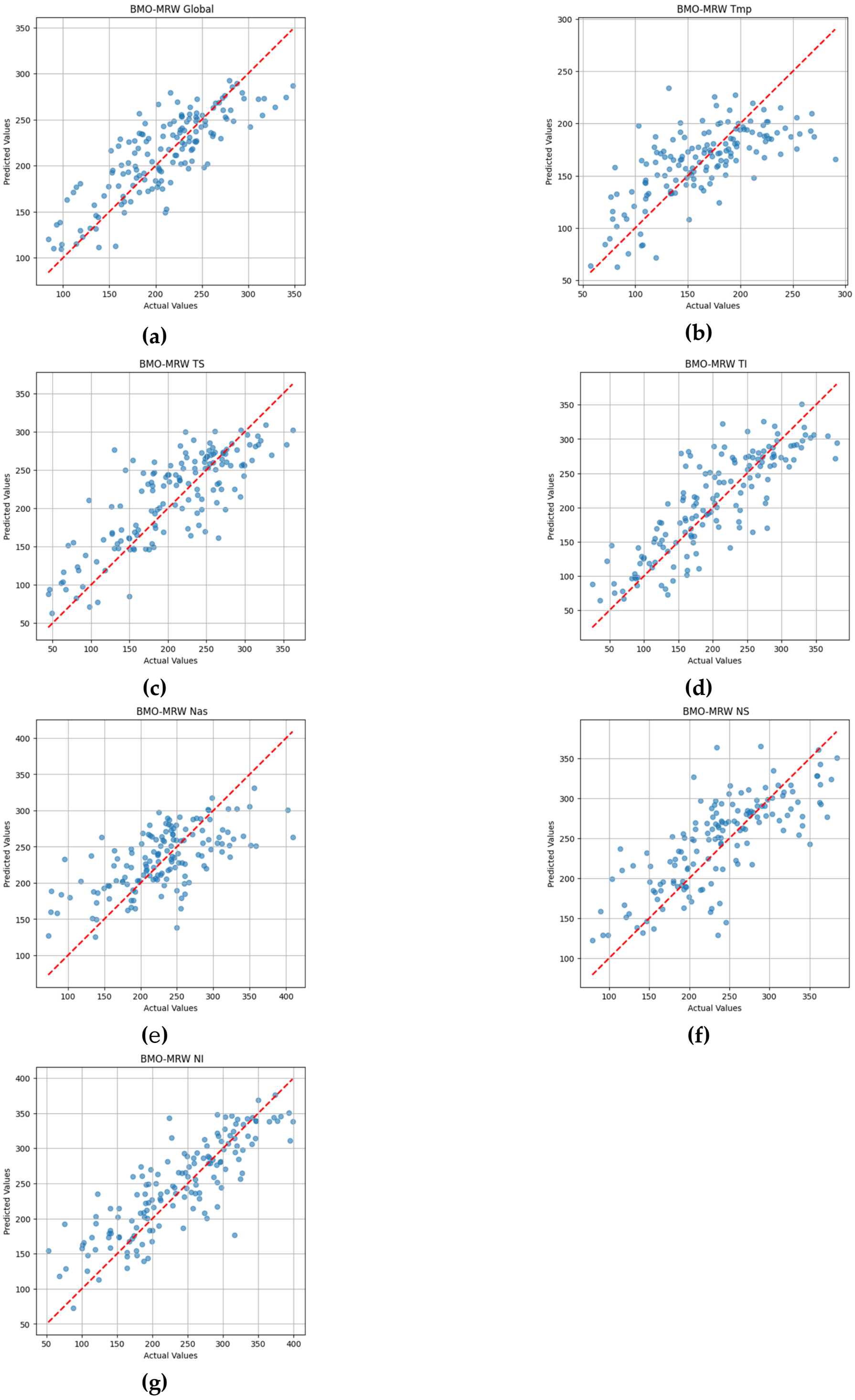

Figure S1. Scatter plots illustrating the comparison between predicted and actual values for the BMO-MRW parameters

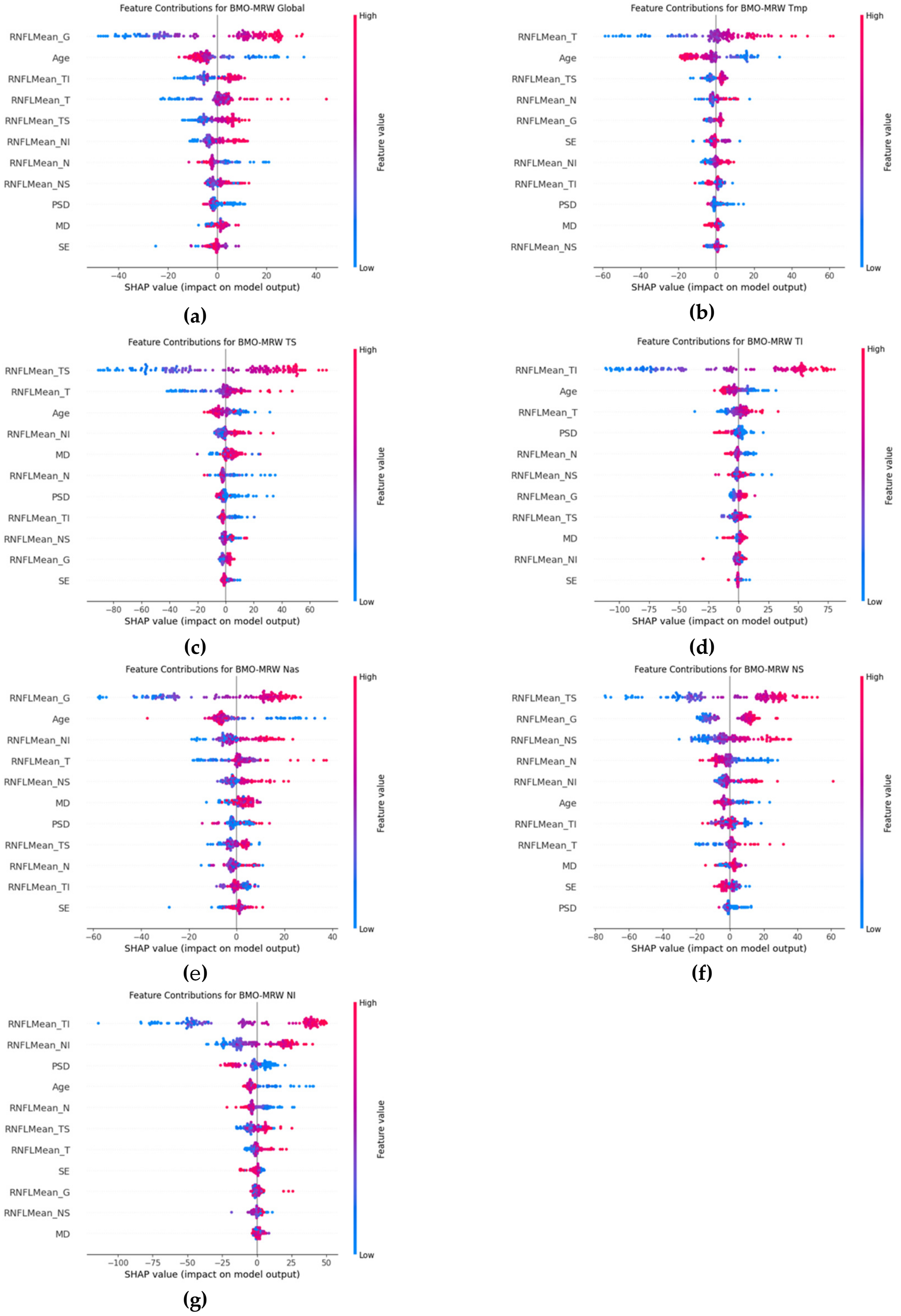

Figure S2. SHAP (SHapley Additive exPlanations) Summary Plots for Feature Contributions to the Prediction of BMO-MRW parameters
